# Measuring Lp(a) particles with a novel isoform-insensitive immunoassay illustrates efficacy of muvalaplin

**DOI:** 10.1016/j.jlr.2024.100723

**Published:** 2024-12-06

**Authors:** Craig A. Swearingen, John H. Sloan, Grace M. Rhodes, Robert W. Siegel, Nico Bivi, Yuewei Qian, Robert J. Konrad, Michael Boffa, Marlys Koschinsky, John Krege, Giacomo Ruotolo, Stephen J. Nicholls, Laura F. Michael, Yi Wen

**Affiliations:** 1 Lilly Research Laboratories, Eli Lilly and Company, Indianapolis, IN, USA; 2 Robarts Research Institute, University of Western Ontario, London, ON, Canada; 3 Victorian Heart Institute, Monash University, Clayton, VIC, Australia

**Keywords:** Lipoprotein (a), antibodies, lipoprotein (a) metabolism, clinical trials, drug therapy, cardiovascular disease, apolipoprotein (a), isoform, muvalaplin, immunoassay

## Abstract

Lipoprotein(a) [Lp(a)] is a cardiovascular risk factor, and there is considerable interest in developing Lp(a)-lowering therapeutics for cardiovascular prevention. Current commercial Lp(a) assays measure total apolipoprotein(a) [apo(a)] and may be insufficient to accurately measure Lp(a) concentrations and determine Lp(a) lowering by a new class of small-molecule Lp(a) formation inhibitors such as muvalaplin. We developed a novel immunoassay that measures only Lp(a) particles. This intact Lp(a) assay demonstrated robust analytical performance, was insensitive to apo(a) isoform size, and correlated with a liquid chromatography–tandem mass spectrometry method. Muvalaplin phase I multiple ascending dose study samples and lepodisiran, a small-interfering RNA that lowers Lp(a), phase I single ascending dose study samples were analyzed using the intact Lp(a) assay and commercial assays. The Lp(a)-lowering efficacy of muvalaplin was underestimated by the commercial assay measuring total apo(a) compared with the intact Lp(a) assay specifically measuring Lp(a) particles. In contrast, the Lp(a)-lowering effect of lepodisiran was clinically comparable between the intact Lp(a) assay and commercial assay. This novel intact Lp(a) assay provides a more accurate approach for the assessment of Lp(a)-lowering agents and the study of Lp(a)-associated risk compared with currently available assays.

Lipoprotein(a) [Lp(a)] is a lipoprotein composed of apolipoprotein(a) [apo(a)] covalently bound to an LDL-like particle containing one molecule of apolipoprotein B-100 (apoB) and is an independent cardiovascular risk factor (1, 2, 3, 4). Apo(a) is encoded by the *LPA* gene, which is only present in the genomes of humans and some non-human primates (5). The *LPA* gene arose from the plasminogen (*PLG*) gene by a gene duplication event late in evolution, where plasminogen kringle domains (K) I, II, and III were lost, and the kringle IV (KIV) domain expanded and diverged to 10 subtypes (KIV1–10) (5). Circulating levels of Lp(a) are determined primarily by hypervariable numbers of KIV type 2 domains (KIV2) as well as by single nucleotide polymorphisms in the *LPA* gene (6). The number of KIV2 repeats per allele varies from 3 to >40 copies, resulting in a large heterogeneity in plasma apo(a) isoform sizes (7, 8). Apo(a) concentrations are generally inversely correlated with apo(a) isoform size and vary widely between individuals (7, 8).

The variable size of apo(a) isoforms poses a challenge for accurately measuring apo(a) (3, 9). Commercial assays are available for Lp(a) clinical measurements, and most are immunoturbidimetric methods that employ anti-apo(a) polyclonal antibodies (10, 11). Therefore, these assays detect both apo(a) in the Lp(a) particle and apo(a) that is not bound to apoB. Another caveat is that binding epitopes of the anti-apo(a) polyclonal antibodies are uncharacterized. Since the polyclonal antibodies likely recognize epitopes that are present on repeated KIV2 domains, the assay results are impacted by variable apo(a) isoform sizes. A common approach to partially overcome this quantitative issue is to apply multi-point calibrators with a range of isoform sizes and concentrations (9, 11). Unless assay calibrators that are used have the same range of apo(a) isoforms as test samples, those with higher numbers of the KIV2 repeated sequences will result in an overestimation in Lp(a) concentrations, whereas those with a smaller number of the KIV2 repeated sequences will result in an underestimation. Colorimetric ELISAs have also been developed. One such assay uses the LP4 monoclonal antibody (mAb) directed to an epitope in apo(a) KIV2 for capture and the LPA-KIV9 mAb targeting a single unique epitope present on KIV9 for detection (12). Like anti-apo(a) polyclonal antibody-based immunoturbidimetric assays, this ELISA also measures total apo(a) as both the capture and detection antibodies target apo(a). Other Lp(a) ELISAs employ a combination of anti-apo(a) and anti-apoB antibodies (8, 13, 14). However, in these assay configurations, the anti-apo(a) antibodies, used either as capture or detection, recognize an epitope in KIV2, which may still result in inaccurate Lp(a) measurements due to sensitivity to apo(a) isoform size (3).

The development of therapeutics that specifically lower Lp(a) levels necessitates reliable and sensitive assays for measuring Lp(a). In healthy subjects, unbound apo(a) represents approximately 5% of total apo(a) (15). Nucleic acid therapeutics, such as pelacarsen, olpasiran, and lepodisiran, inhibit the production of apo(a) by degrading the *LPA* messenger RNA (mRNA) (16, 17, 18). In these scenarios, the detection of unbound apo(a) will contribute very little to Lp(a) measurement. In contrast, muvalaplin, a small-molecule inhibitor of Lp(a) formation, reduces circulating Lp(a) levels by binding to apo(a) KIV7–8 domains and preventing interaction of apo(a) with apoB on LDL particles (19, 20). Because of muvalaplin’s mechanism of action, we hypothesize that current commercial Lp(a) assays may underestimate the magnitude of Lp(a) lowering by muvalaplin because they measure total apo(a) and may detect apo(a)–muvalaplin complexes in circulation. To test this hypothesis, we developed an isoform-insensitive, Lp(a) particle-specific electrochemiluminescent (ECL) immunoassay, referred to as intact Lp(a) assay hereafter. We compared Lp(a) measurements performed with this intact Lp(a) assay and commercial immunoturbidimetric assays using phase I trial samples of lepodisiran, an Lp(a)-lowering small interfering RNA (siRNA), and muvalaplin, an Lp(a) formation disrupter.

## Materials and Methods

### Materials

Randox Lp(a) calibrator set (Cat# LP3404) and Randox Lp(a) assay reagents (Cat# LP2757) were purchased from Randox Laboratories Ltd. Several vials of Randox Lp(a) calibrator #3 were reconstituted as directed, pooled, aliquoted, and stored at −80°C. Lp(a) and ApoB, purified from human plasma, were purchased from Athens Research and Technology. Human plasminogen protein was purchased from R&D Systems. Heterophilic blocking reagent-1 (Cat# 3KC533) was purchased from Scantibodies Laboratory, Inc, aliquoted, and stored at −20°C.

To produce recombinant human apo(a), a nucleotide sequence encoding human apo(a) (NP_005568.2) was inserted into a mammalian expression vector containing a cytomegalovirus promoter. Protein expression was performed through transient transfection in HEK293 cells that were cultured in serum-free media. Culture media were harvested 5 days post-transfection, and human apo(a) protein was purified using L-lysine affinity chromatography followed by size-exclusion chromatography (SEC). To produce various KIV domains (for instance, KIV2 and KIV7-8-9-10), each nucleotide sequence encoding the KIV domain of interest was inserted into a pET21a *Escherichia coli* expression vector. Bacterial BL21(DE3) was used as the expression host. KIV domain proteins were expressed as inclusion bodies and refolded using the rapid-dilution method followed by SEC. Protein concentrations were determined by measurement of absorbance at 280 nm (A_280_).

A set of 40 serum samples was acquired from the US Centers for Disease Control and Prevention (CDC). These samples are from the Apolipoprotein Standardization Program, part of the CDC Clinical Standardization Programs. Samples are non-pooled human sera from single donors obtained by the C37 protocol. Lp(a) orientation values were assigned by an isotope dilution liquid chromatography with tandem mass spectrometry (LC-MS/MS) method, and Lp(a) isoform sizes (total KIV number) were determined by gel electrophoresis and western blotting per the sample report provided by the CDC. In addition, a set of 60 healthy human serum samples were purchased from BioIVT (Hicksville) for assay development.

### Anti-apo(a) capture antibody and anti-apolipoprotein B-100 (apoB) detection antibody

To generate anti-apo(a) antibodies that do not recognize KIV2, the recombinant apo(a) KIV7-8-9-10 protein was used to immunize mice. Clones were screened for binding to recombinant KIV7-8-9-10 protein and counter-screened for binding to recombinant KIV2 protein. For clones of interest, the variable heavy and variable light gene sequences were determined from extracted RNA. Variable domains were transferred into separate murine constant region expression vectors followed by transfection into Chinese hamster ovarian cells for antibody production. Antibodies were purified using protein A chromatography. The binding specificity of anti-apo(a) antibodies was determined with bio-layer interferometry using Octet Red96 (ForteBio). Antibodies were diluted to 10 μg/ml in running buffer (HEPES buffered saline + 0.1% BSA), immobilized onto anti-mouse immunoglobulin G (IgG) biosensor tips, and washed in running buffer. The tips were then incubated with various recombinant apo(a) KIV domain-containing proteins, recombinant apo(a), apo-B, and Lp(a) for 300 s. Dissociation was observed after transfer of the tips to the running buffer.

Anti-apo(a) capture antibody was labeled with biotin, and anti-apoB monoclonal detection antibody (produced internally) was labeled with ruthenium. Briefly, EZ-Link™ Sulfo-NHS-LC-Biotin at 10 mM (Thermo Fisher Scientific, Cat# A39257) was combined with anti-apo(a) capture antibody at a 10:1 M ratio and incubated on ice for 2 h. For ruthenium labeling, 3 mM MesoScale Discovery (MSD) SULFO-TAG NHS-Ester (MSD, Cat# R91AN) was mixed with anti-apoB detection antibody at a 12:1 M ratio and incubated on ice for 2 h. Labeling reactions were stopped by the addition of a 1/10 volume of 1 M Tris-HCl, pH 7.5. Labeled antibodies were concentrated using an Amicon filter device (30 KDa molecule weight cut-off) and then purified by SEC. The final mass concentration was determined by bicinchoninic acid assay, and an equal volume of glycerol was added before storage at −20°C.

### Intact Lp(a) immunoassay

MSD Gold streptavidin plates (MSD, Cat#: L15SA-1) were washed three times with TBS – 0.05% Tween 20 (TBS-T). Plates were blocked with 200 μl TBS + 1% BSA for 1 h at room temperature (RT). Plates were washed, and 50 μl biotinylated anti-apo(a) capture antibody at 1 μg/ml in TBS-T + 0.1% BSA was added to the plates. After 1 h incubation, plates were washed, and 50 μl 3-fold serially diluted standards (top standard at 0.925 nM or 0.5 mg/dl, diluted from level 3 Randox calibrator) or serum samples that were diluted in assay dilution buffer (50 mM pH 7.5 HEPES, 150 mM NaCl, 1% Triton X-100, 5 mM EDTA, 5 mM EGTA, 0.1% BSA, and 100 μg/ml heterophilic blocking reagent-1) were added to the plates for a 1 h incubation at RT. Standards, controls, and samples were tested in duplicate. Plates were washed again, and 50 μl of ruthenium-labeled anti-apoB detection antibody at 1 μg/ml in detection buffer (50 mM pH 7.40 HEPES, 150 mM NaCl, 1% Triton X-100, 5 mM EDTA, 5 mM EGTA, and 0.1% BSA) were added followed by a 1 h incubation at RT. After the final washes, 150 μl of MSD read buffer was added. ECL signals were recorded in an MSD Quick Plex SQ 120 reader (MSD). FourPL algorithm standard curve fitting and calculation of sample concentrations were performed in the MSD Discovery Workbench 4.0 Analysis Software. The theoretical standard concentrations could be assigned in either nM or mg/dl to calculate sample concentrations in nM or mg/dl, respectively. The average of the two technical replicates was calculated as the reportable result for each sample.

### Apo(a) spike experiment

Recombinant apo(a) was diluted in TBS buffer to prepare a series of 10X spikes. Five μL of 10X apo(a) spikes were mixed with 45 μl normal human serum to prepare 1X apo(a) spike samples at different concentrations. The apo(a) spike samples were analyzed using the intact Lp(a) assay described above and the Randox assay following the manufacturer’s protocol.

### Assay development and qualification

The intact Lp(a) assay was qualified to measure Lp(a) levels in human serum and plasma samples. Fit-for-purpose assay development and qualification included standard curve assessment, minimal required dilution (MRD), lower limit of quantification (LLOQ), dilutional linearity, intra-assay precision, inter-assay precision, stability, and matrix comparability. Three levels of controls – high positive control, mid positive control, and low positive control – were prepared by screening and aliquoting three individual serum samples containing high, mid, and low levels of Lp(a), respectively. For standard curve assessment, 64 sets of standard curves were collected over 30 independent runs. The mean and % coefficient of variation (CV) of the ECL unit signals and back-calculated values at each standard level were calculated. The acceptance criteria were <25% CV for standards above LLOQ and ≤30% CV at LLOQ. The percent change of the back-calculated value from the theoretical value at each standard level was calculated, and the acceptance criterion was defined as within ±25%. Two levels of MRD (2,500 and 10,000) were evaluated by determining spike recovery in serum samples, and the criterion was within ±30% of the matched spike in a buffer. LLOQ was assessed by thawing and diluting the mid-positive control by 1:2, 1:4, 1:8, 1:16, 1:32, 1:64, 1:128, 1:256, 1:512, 1:1,024, and 1:2,048 in dilution buffer. Aliquots were stored at −80°C and analyzed on four separate plates over 2 days to generate four reportable results (back-calculated value). Mean and %CV were calculated. The lowest back-calculated value that was within the detection range and with %CV <30% was defined as the LLOQ.

To evaluate dilutional linearity, a human serum sample containing a high level of Lp(a) was diluted to 1:20 followed by 11 subsequent 2-fold serial dilutions. Each serially diluted sample was analyzed directly in the intact Lp(a) assay without undergoing the procedural sample dilution, i.e., MRD. %CV for samples that were within the detection range was calculated. Dilutional linearity was defined as dilutions above LLOQ that exhibited <25% CV. Twelve sets of positive controls were analyzed on one plate, and the mean signal and %CV of each control were calculated to evaluate intra-assay precision. To assess inter-assay precision, the mean signal and %CV of 61 reportable results (grand mean on a plate) across 30 independent experimental runs were calculated. Acceptance criteria for intra-assay precision and inter-assay precision of the three controls were set at <25% CV. To assess stability, aliquots of frozen controls were stored at room temperature (4, 24, and 48 h) and 2–8°C (4, 24, 48 h), and subjected to four, six, and eight freeze/thaw cycles before analysis. Freshly thawed controls were tested on the same plate, and their levels were used as the baseline to calculate percent change. Stability was considered acceptable if the percent change was ≤30%. Finally, donor-matched serum and EDTA plasma samples were analyzed to assess matrix comparability.

### Clinical trial samples and sample analysis

Samples from the lepodisiran phase I single ascending dose (SAD) study and muvalaplin phase I multiple ascending dose (MAD) study were analyzed. The clinical study design and protocol, informed consent, and study population have been described (18, 19). The study protocols were approved by independent ethics committees and were carried out in accordance with the Declaration of Helsinki and in compliance with current regulations and standards of Good Clinical Practice. All participants provided written informed consent. Previously, Lp(a) measurements of lepodisiran SAD samples and muvalaplin MAD samples were performed using the Roche Cobas analyzer (Roche assay) and Siemens Atellica CH Analyzer (Siemens assay), respectively. The Roche assay used the Lp(a) assay reagents and 5-level Lp(a) calibrators from Randox Laboratories Ltd, and the Siemens assay used Atellica CH 5-level Lp(a) calibrators. In the current study, stored lepodisiran SAD samples and muvalaplin MAD samples were analyzed by the intact Lp(a) assay. All samples were tested in duplicate. The average of the two technical replicates was calculated as the reportable result for each sample. The reportable result was accepted when the sample, standards, and positive controls on the plate all met pre-defined acceptance criteria.

### Statistical analysis

Statistical analysis was conducted on the data from the CDC Apolipoprotein Standardization Program samples to assess the relationship between the intact Lp(a) assay and the LC-MS/MS method and to assess the intact Lp(a) assay’s relationship to isoform size. Statistical analysis was also conducted on data from lepodisiran and muvalaplin phase I studies to assess the relationship between the intact Lp(a) assay and immunoturbidimetric assays in the presence of such treatments. For each analysis, only samples evaluated with both assays under study were included in the calculations. The relationships between assays were assessed via Pearson’s correlation coefficient, correlation plots with a line of best fit from weighted Deming regression, and Bland–Altman plots. Mean bias was calculated as the mean absolute difference between assay measurements for paired samples. Percent bias was calculated as the absolute difference divided by the mean for each paired sample, averaged over all paired samples. The mean absolute Lp(a) level and the mean percent change from baseline in Lp(a) were calculated for muvalaplin and lepodisiran samples across study days. Sample data were log-transformed before such calculations, and the delta method was implemented to back-transform data to the original scale. Two-sided *P*-values were computed via Student’s *t* test on log-transformed data to compare the mean percent change from baseline between assays. Given the exploratory nature of analyses, no multiple testing adjustment was performed.

## Results

### Characterization and development of anti-apo(a) antibody and intact Lp(a) assay

**Fig. 1:**
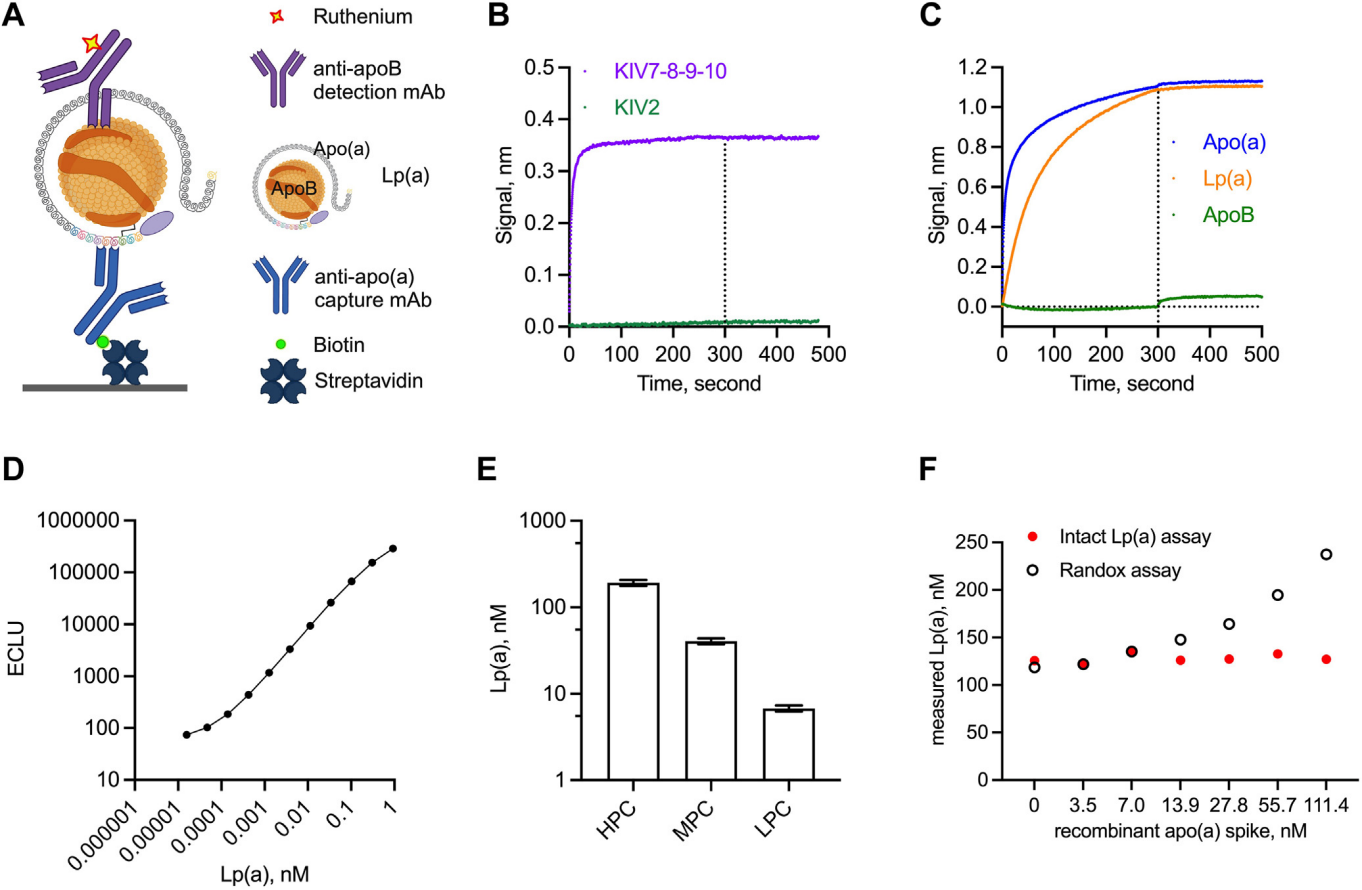
Intact lipoprotein(a) [Lp(a)] assay and the anti-apolipoprotein(a) [apo(a)] capture antibody. A: Schematic of the intact Lp(a) assay using anti-apo(a) capture antibody and anti-apoB detection antibody. B: Binding specificity of the anti-apo(a) capture antibody. C: Binding to apo(a), Lp(a), and apoB by the anti-apo(a) capture antibody. D: Standard curve of the intact Lp(a) assay. Mean and standard deviation (SD) were plotted at each standard level. E: Positive control precision of the intact Lp(a) assay. Mean and SD were plotted for each positive control (PC). F: Comparison of recombinant apo(a) detection by the intact Lp(a) assay and Randox assay. apoB, apolipoprotein B-100; ECLU, electrochemiluminescent unit; HPC, high positive control; KIV, apo(a) Kringle IV domain; LPC, low positive control; mAb, monoclonal antibody; MPC, mid positive control. Pane lA was created with BioRender.com.

Using an anti-apo(a) antibody that does not recognize KIV2 is critical to creating an Lp(a) assay with isoform insensitivity and combining anti-apo(a) and anti-apoB antibodies in a sandwich format can enable specific detection of intact Lp(a) particles (8, 12, 13, 21). Anti-apo(a) antibodies were discovered from immunization with apo(a) KIV7-8-9-10 for use as a capture antibody to pair with an anti-apoB detection antibody (Fig. 1A). The anti-apo(a) capture antibody selected for the intact Lp(a) assay bound to KIV7-8-9-10 but not to KIV2 (Fig. 1B). The selective binding to apo(a) domains was further studied by immunoblotting, which confirmed that the capture antibody bound to different apo(a) domain proteins, except KIV types 1-2-3-4 (supplemental Fig. S1). This capture antibody showed desired binding to recombinant apo(a) protein and Lp(a) particles, but not to apoB (Fig. 1C). When paired with the ruthenium-labeled anti-apoB detection antibody, the assay exhibited excellent dynamic range (0.000016–0.925 nM) and standard curve and quality control precision (<10% CV at all levels) (Fig. 1D, E, Table 1, and supplemental Table S1). Unlike apo(a), a quick off rate was observed for plasminogen by the anti-apo(a) antibody and plasminogen did not produce signals in the intact Lp(a) assay (supplemental Fig. S2).

**Table 1:** Summary of assay performance parameters

Assay Parameter	Value
Standard curve	Back-calculated value within ±5% of theoretical value
	ECLU at all concentrations <10% CV
	Back-calculated value < 5% CV for first 10 standard concentrations, and <15% CV for the 11^th^ standard concentration
Sample dilution (MRD)	1:2,500 or 1:10,000
LLOQ	0.69 nM (in the sample before dilution)
Dilutional linearity	1:320–1:40,960
Intra-assay precision (% CV)	HPC: 3.0
	MPC: 2.3
	LPC: 4.9
Inter-assay precision (% CV)	HPC: 8.1
	MPC: 7.6
	LPC: 8.3
Stability	Room temperature: 48 h
	2–8°C: 48 h
	Freeze-thaw: Eight cycles
Matrix	<10% CV between donor-matched serum and EDTA plasma

CV, coefficient of variation; ECLU, electrochemiluminescent unit; EDTA, ethylenediaminetetraacetic acid; HPC, high positive control; LLOQ, lower limit of quantification; LPC, low positive control; MPC, mid positive control; MRD, minimal required dilution.

To demonstrate that this assay detects only Lp(a) particles but not unbound apo(a), serially diluted recombinant apo(a) protein was spiked into normal human serum and spiked human serum samples were analyzed by the intact Lp(a) assay and Randox assay. At 0 ng/ml of spiked apo(a), both assays measured endogenous Lp(a) levels and were comparable (Fig. 1F). A clear dose-response curve of spiked apo(a) was detected by the Randox assay. In contrast, measurements by the intact Lp(a) assay remained flat, indicating that the intact Lp(a) assay did not detect the spiked apo(a) (Fig. 1F). The intact Lp(a) assay was adequately qualified, and demonstrated desired dilutional linearity (1:320–1:40,960), sensitivity (0.69 nM), stability, and matrix compatibility (Table 1). Lp(a) levels in 60 individual healthy human volunteer samples were measured and showed <10% CV for 56 samples and <20% CV for the remaining four samples between two independent measurements on two separate days (supplemental Table S2). In addition, binding of the anti-apo(a) antibody to apo(a) was not interfered with by muvalaplin (supplemental Fig. S3). Lp(a) measurements in serum samples of different Lp(a) levels were also not interfered with by muvalaplin (supplemental Table S3). These results collectively indicate that the intact Lp(a) assay is robust, sensitive, isoform independent, and Lp(a) particle specific.

### Correlation with liquid chromatography with tandem mass spectrometry method

**Fig. 2:**
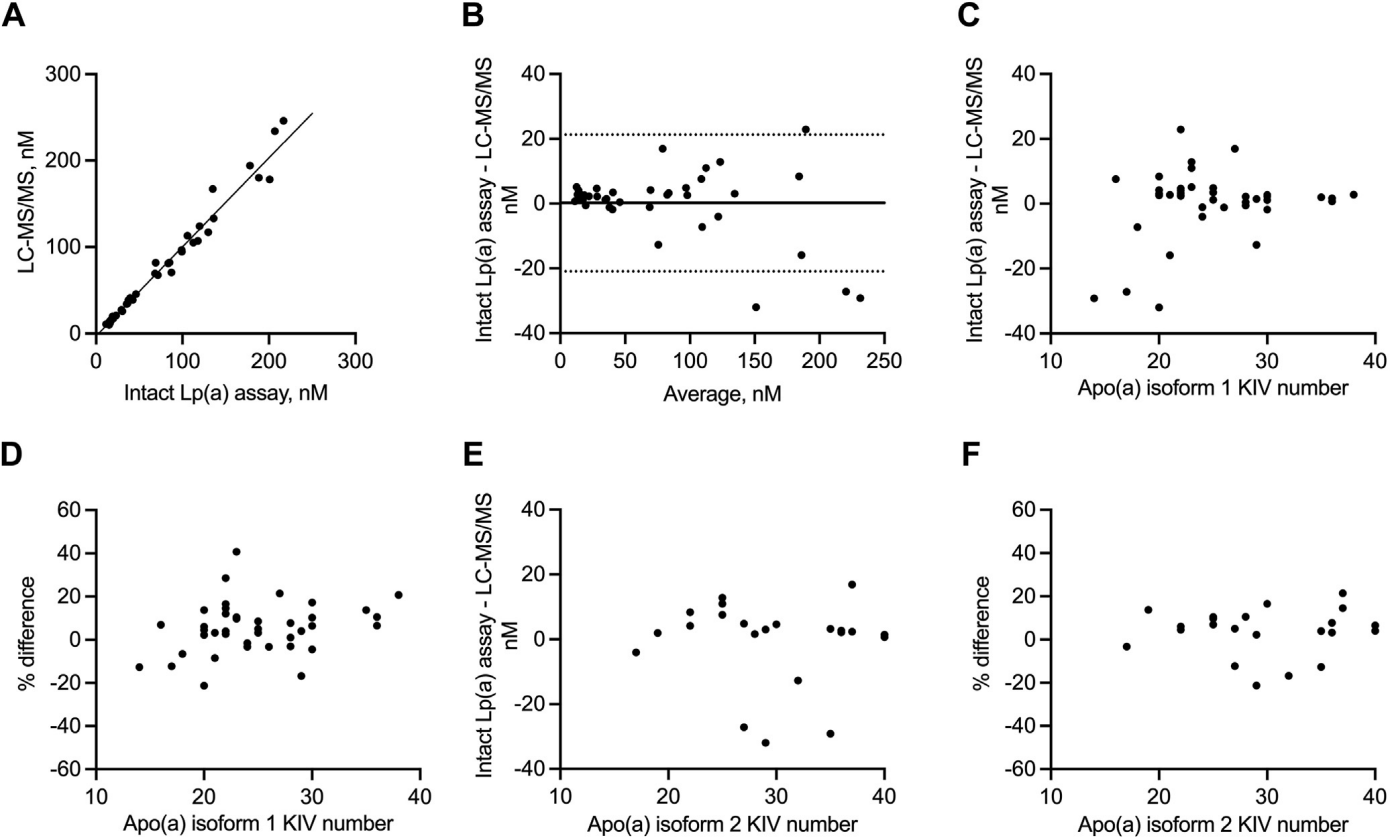
Correlation between intact Lp(a) assay and LC-MS/MS method. A: X–Y correlation plot for the intact Lp(a) assay and LC-MS/MS method. Solid line is the weighted Deming regression line of best fit (slope = 1.03, Y-intercept = −2.89). B: Bland–Altman correlation plot for the intact Lp(a) assay and LC-MS/MS method. Solid line represents the mean bias (0.19 nM), and dotted lines are bias ± 1.96 × standard deviation. C: Difference between the intact Lp(a) assay and LC-MS/MS method versus apo(a) isoform 1 size by KIV number. D: % difference between the intact Lp(a) assay and LC-MS/MS method versus apo(a) isoform 1 size by KIV number. E: Difference between the intact Lp(a) assay and LC-MS/MS method versus apo(a) isoform 2 size by KIV number. F: % difference between the intact Lp(a) assay and LC-MS/MS method versus apo(a) isoform 2 size by KIV number. apo(a), apolipoprotein(a); KIV, kringle IV; LC-MS/MS, liquid chromatography with tandem mass spectrometry; Lp(a), lipoprotein(a).

The Apolipoprotein Standardization Program at the CDC provides 40 individual human serum samples as a kit. Lp(a) concentrations were characterized using an LC-MS/MS method, and the apo(a) KIV numbers were determined by gel electrophoresis and western blotting by the CDC. Total KIV numbers vary from 14 to 38 for isoform 1 and from 17 to 40+ for isoform 2. However, only 22 samples have their KIV numbers of isoform 2 determined, possibly due to low or no expression of the second isoform and assay sensitivity (6). For analysis purposes, isoform numbers >40 were imputed as 40, as exact values are unknown. Samples were analyzed by the intact Lp(a) assay to investigate the correlation with LC-MS/MS and confirm isoform insensitivity. The correlation of measured Lp(a) concentrations between the intact Lp(a) and LC-MS/MS method was shown by the X–Y correlation plot and Bland–Altman plot (Fig. 2A, B). The Pearson correlation coefficient r was 0.99. The mean bias was 0.19 nM (5.5%), and 1.96 × standard deviation (SD) limits of agreement were −20.91 to 21.30 nM. The relationship between the difference (and % difference) of the two assays and isoform KIV numbers was examined (Fig. 2C–F). Simple linear regression indicates that KIV isoform 1 number explains only 5.9% of the variability in the difference (*r*^2^ = 0.059) and 6.5% of the variability in the percentage difference (*r*^2^ = 0.065) between assays, whereas KIV isoform 2 number explains only 0.3% of the variability in the difference (*r*^2^ = 0.003) and 0.2% of the variability in the percentage difference (*r*^2^ = 0.002). The results indicate that the intact Lp(a) assay was not meaningfully sensitive to apo(a) isoforms.

### Lepodisiran phase I single ascending dose study

**Fig. 3:**
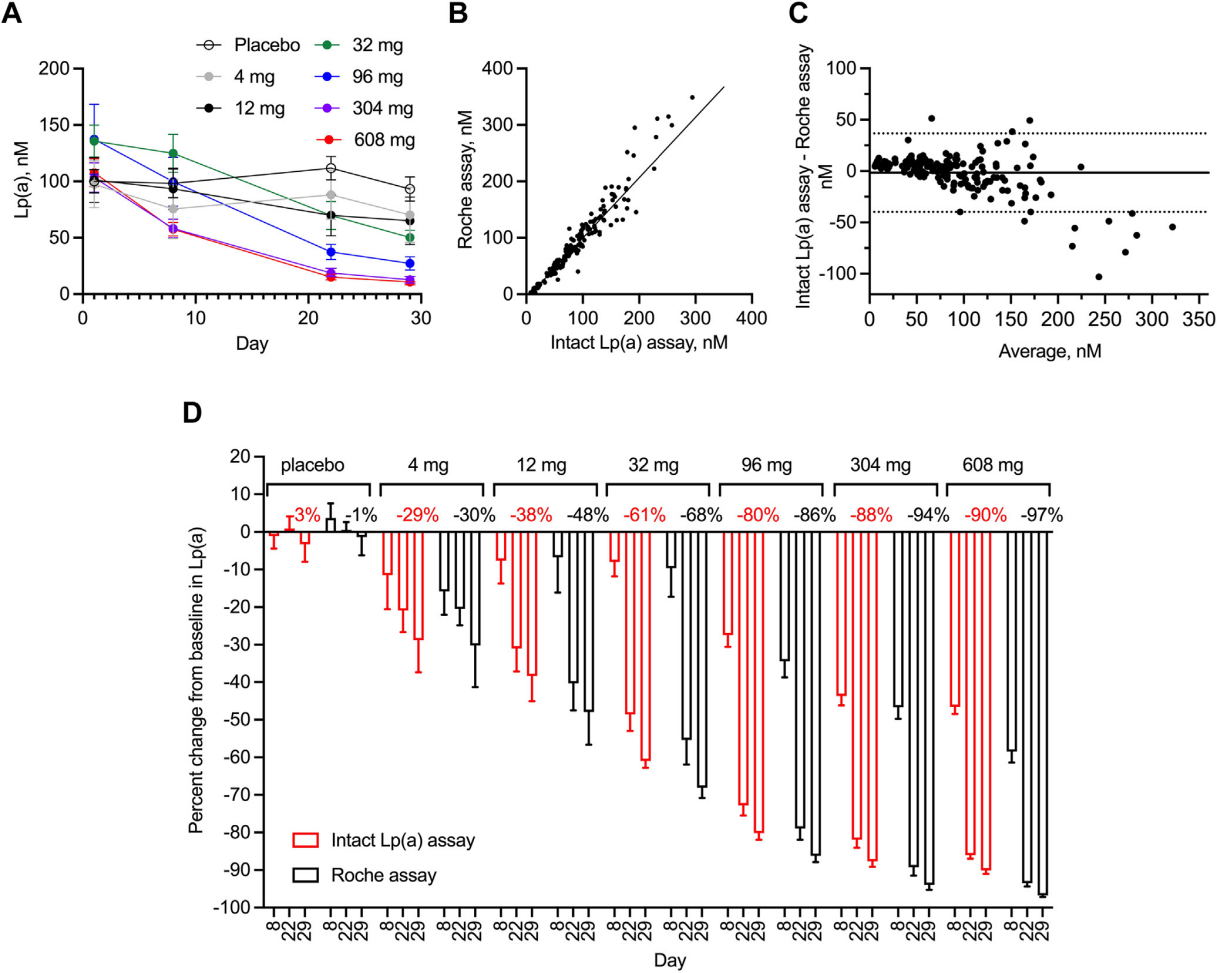
Comparison and correlation between intact Lp(a) assay and Roche assay using lepodisiran samples. A: Concentrations of Lp(a) over time by treatment groups measured by the intact Lp(a) assay. B: X–Y correlation plot for the intact Lp(a) assay and Roche assay using baseline and post-baseline samples from placebo- and lepodisiran-treated groups. The solid line is the weighted Deming regression line of best fit (slope = 1.07, Y-intercept = −7.35). C: Bland–Altman correlation plot for the intact Lp(a) assay and Roche assay using baseline and post-baseline samples from placebo- and lepodisiran-treated groups. The solid line represents the mean bias (−1.58 nM), and the dotted lines are biased ± 1.96 × standard deviation. D: Percent change from baseline calculated from Lp(a) concentrations measured by intact Lp(a) assay or Roche assay. Lp(a), lipoprotein(a); SAD, single ascending dose.

Lepodisiran is a N-acetylgalactosamine (GalNAc)-conjugated siRNA that lowers Lp(a) by degrading the *LPA* mRNA (18). Because lepodisiran reduces Lp(a) by silencing the expression of apo(a), we hypothesized that the Lp(a) levels measured in lepodisiran-treated participants by the intact Lp(a) assay and commercial immunoturbidimetric assays should be comparable. Indeed, dose-dependent Lp(a) reduction was observed when Lp(a) was measured by the Roche immunoturbidimetric assay and the intact Lp(a) assay (Fig. 3A). The assay results correlated well as shown by the X–Y correlation plot, and at levels up to 200 nM Lp(a) by the Bland–Altman plot (Fig. 3B, C). The Pearson correlation coefficient r was 0.97. The mean bias was −1.58 nM (11.7%), and 1.96 × SD limits of agreement were −39.77 to 36.60 nM. Percent change from baseline in Lp(a) was calculated for each assay, and there was good agreement between the intact Lp(a) assay and Roche immunoturbidimetric assay (Fig. 3D).

### Muvalaplin phase I multiple ascending dose study

Muvalaplin is an oral small-molecule inhibitor that lowers Lp(a) by binding to KIV7-8 in apo(a) and blocking the formation of Lp(a) particles (19, 20). This mechanism of action may lead to a transient presence of muvalaplin-bound apo(a) in the circulation that is detectable by commercial immunoturbidimetric assays that rely solely on anti-apo(a) polyclonal antibodies. We hypothesized that the Lp(a)-lowering efficacy of muvalaplin may be underestimated by immunoturbidometric assays and that the intact Lp(a) assay would more accurately measure Lp(a) lowering.

**Fig. 4:**
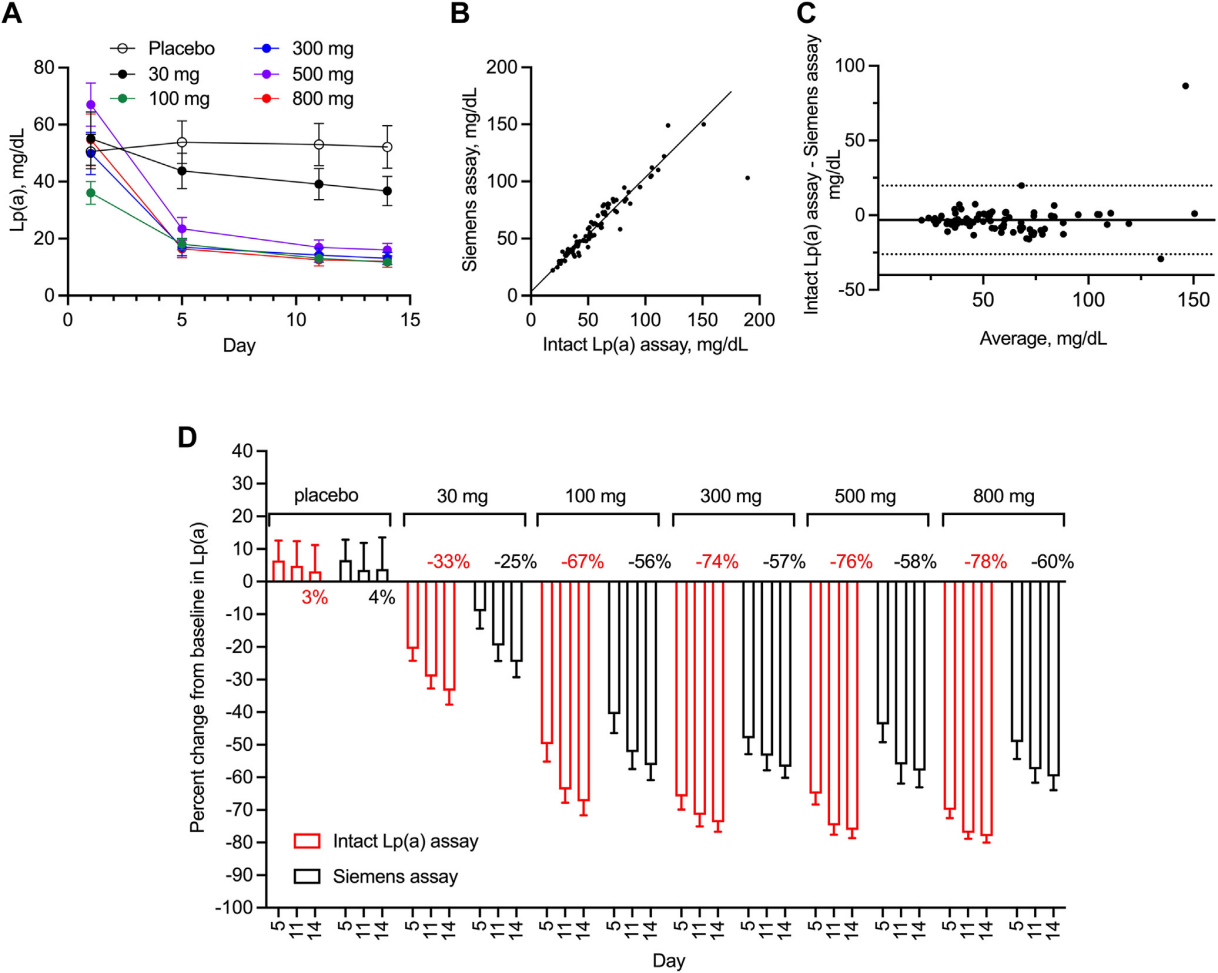
Comparison and correlation between intact Lp(a) assay and Siemens assay using muvalaplin samples. A: Concentrations of Lp(a) over time by treatment groups measured by the intact Lp(a) assay. B: X–Y correlation plot for the intact Lp(a) assay and Siemens assay using baseline and post-baseline samples from the placebo group and baseline samples only from muvalaplin-treated groups. Solid line is the weighted Deming regression line of best fit (slope = 1.0, Y-intercept = 3.59). C: Bland–Altman correlation plot for the intact Lp(a) assay and Siemens assay using baseline and post-baseline samples from the placebo group and baseline samples only from muvalaplin-treated groups. The solid line represents the mean bias (−3.18 mg/dl), and the dotted lines are bias ± 1.96 × standard deviation. D: Percent change from baseline calculated from Lp(a) concentrations measured by intact Lp(a) assay or Siemens assay. Lp(a), lipoprotein(a); MAD, multiple ascending dose.

Since Lp(a) was previously measured in mg/dl by the Siemens immunoturbidimetric assay, sample results were exported in mg/dl by inputting the standards in mg/dl concentration units for the intact Lp(a) assay to enable correlation analysis in the same concentration unit. In agreement with the Siemens assay results, muvalaplin lowered Lp(a) dose-dependently when Lp(a) levels were measured by the intact Lp(a) assay (Fig. 4A). Because of the hypothesized presence of unbound apo(a) after muvalaplin treatment and the difference in specificity between the two assays, a correlation was examined using samples from participants and/or time points without muvalaplin, which included all time points from placebo-treated study participants and baseline samples from muvalaplin-treated study participants. A strong correlation was demonstrated between the two assays (Fig. 4B, C). The Pearson correlation coefficient r was 0.92. The mean bias was −3.18 mg/dl (−7.1%), and 1.96 × SD limits of agreement were −26.10 to 19.74 mg/dl. More importantly, the intact Lp(a) assay indeed indicated that muvalaplin had a stronger Lp(a)-lowering effect than previously determined by the Siemens immunoturbidimetric assay (Fig. 4D). By day 14, muvalaplin reduced Lp(a) particles by 33%, 67%, 74%, 76%, and 78% at 30 mg, 100 mg, 300 mg, 500 mg, and 800 mg doses, respectively, measured by the intact Lp(a) assay, compared with 25%, 56%, 57%, 58%, and 60%, respectively, measured by the Siemens immunoturbidimetric assay (Fig. 4D). Statistical significance was confirmed at each timepoint (days 5, 11, and 14) for all muvalaplin-treated groups (*P* < 0.05), whereas no statistical difference in Lp(a) lowering between the two assays was found for the placebo group (*P* = 0.98 for day 5, *P* = 0.74 for day 11, and *P* = 0.80 for day 14).

## Discussion

Effective Lp(a) assays are essential for accurate evaluation of the role of Lp(a) in a range of forms of cardiovascular disease and for clinical development of Lp(a)-targeted therapeutics. A desired Lp(a) assay should be insensitive to Lp(a) isoforms, specific to Lp(a) particles, report results in nanomoles per liter (nmol/L, nM), and use standards that are traceable to internationally accepted reference materials (3). With these performance parameters and features in mind, we developed a robust and sensitive Lp(a) assay that is isoform-independent and Lp(a) particle-specific. The anti-apo(a) capture antibody likely recognizes a common epitope that is present in KIV7, KIV8, KIV9, and KIV10, but not in KIV2 or KIV1-2-3-4 (Fig. 1B and supplemental Fig. S1), which is essential for isoform insensitivity. Unlike the variable sizes of KIV2, the KIV7-8-9-10 domain is the same in different apo(a) isoforms, therefore, this anti-apo(a) antibody will bind all apo(a) isoforms equally and should not introduce bias in assay results. The assay uses an anti-apoB mAb for detection, therefore the assay will not detect unbound apo(a) (Fig. 1F). An anti-apo(a) capture and anti-apoB detection configuration may be advantageous compared with an anti-apoB capture and anti-apo(a) detection configuration. This is because the anti-apoB capture and anti-apo(a) detection configuration may be subject to potential saturation and biased immobilization of high concentrations of apoB-containing particles and different species of apoB-containing particles in circulation.

The Randox Lp(a) assay is based on the Denka Seiken Lp(a) assay and is traceable to the World Health Organization/International Federation of Clinical Chemistry SRM 2B reference material (10). Randox calibrators are commercially available from Randox Laboratories Ltd., and they are in the form of lyophilized human serum containing Lp(a) with concentrations assigned both in mg/dl and nM. The third-level Randox calibrator was chosen as the standard for an 11-point standard curve. In healthy subjects, unbound apo(a) contributes minimally to total apo(a). Therefore, using values assigned as total apo(a) should not cause significant deviations. Indeed, the measured concentration of the third level Randox calibrator as an unknown by the intact Lp(a) assay was 62.6 nM, which was within 0.8% of the assigned value of 63.1 nM that we used. This notion is further supported by the correlation of the intact Lp(a) assay with LC-MS/MS assay and commercial total apo(a) assays using normal human serum samples and non-muvalaplin-treated patient samples (Fig. 2, Fig. 3, Fig. 4). The intact Lp(a) assay uses ECL as a readout and demonstrates the desired sensitivity, stability, and precision (Table 1).

In addition to analytical performance, the isoform insensitivity of the intact Lp(a) assay was carefully examined. The intact Lp(a) assay exhibited excellent agreement (Pearson correlation coefficient r = 0.99, mean bias = 0.19 nM) with an LC-MS/MS method using 40 human samples of diverse Lp(a) isoform sizes and concentrations (Fig. 2A, B). More importantly, the difference and percentage difference between the intact Lp(a) assay and LC-MS/MS method was not meaningfully dependent on isoform sizes by KIV repeat number (Fig. 2C–F). Correlation analysis between the intact Lp(a) assay and commercial assays (Roche and Siemens assays) revealed good agreement on par with correlations reported between two commercial assays or between a commercial assay and ELISA (10, 11, 14). It is noted that there were larger biases at >200 nM Lp(a) concentrations between the intact Lp(a) assay and Roche assay (Fig. 3C). Similar observations have been made between two commercial assays and between the Randox assay and ELISA (11, 14). Possible explanations include different dilution factors, different affinities and specificities of antibodies, and detection of unbound apo(a).

The intact Lp(a) assay was deployed to determine the Lp(a)-lowering effect of two therapeutics with distinctive mechanisms of action: lepodisiran and muvalaplin. Lepodisiran is an siRNA that knocks down the expression of apo(a) by degrading the *LPA* mRNA, whereas muvalaplin is a small molecule that inhibits Lp(a) particle formation by binding to KIV7-8 of nascent apo(a) (18, 19). Anti-apo(a) polyclonal antibody-based commercial immunoassays cannot differentiate Lp(a) particles from unbound apo(a), which may underestimate the Lp(a)-lowering effect of muvalaplin due to the presence of transient apo(a)-muvalaplin complexes. Indeed, an additional absolute 17%–18% Lp(a) lowering was observed using the intact Lp(a) assay compared with the Siemens assay at doses of 300 mg, 500 mg, and 800 mg (Fig. 4D). Interestingly, there was a difference of absolute 6%–7% in Lp(a) lowering by lepodisiran between the intact Lp(a) assay and Roche assay at higher doses (Fig. 3D). This could possibly be explained by different assay measurements at high Lp(a) levels versus at low Lp(a) levels. At high Lp(a) levels, corresponding to baseline samples, the intact Lp(a) assay generally reported lower Lp(a) concentrations than did the Roche assay. Conversely, at low Lp(a) levels, corresponding to post-treatment samples, the intact Lp(a) assay generally reported higher Lp(a) concentration since the intact Lp(a) assay has better sensitivity than the Roche assay (0.69 nM vs. 6.6 nM). In the case of the commercial assay, this results in values below the LLOQ of the assay being arbitrarily designated at one-half the LLOQ, resulting in possible overestimation of the percent reduction achieved. For instance, for a sample with approximately 6 nM Lp(a), the intact Lp(a) assay will be able to measure 6 nM because this concentration is above the LLOQ, whereas 3.3 nM (LLOQ/2) would be the imputed value for the commercial assay.

An ongoing discussion in the field involves how much Lp(a) lowering is needed for therapeutic benefit/outcome (22, 23, 24, 25). Burgess *et al.* showed that therapeutic benefits may be proportional to absolute Lp(a) reduction and concluded that a significant reduction of approximately 100 mg/dl may be required to produce a clinically meaningful reduction in cardiovascular disease risk (25). However, Madsen *et al.* subsequently suggested that short-term (5 years) Lp(a) lowering of 50 mg/dl (105 nM) may translate to 20% risk reduction in cardiovascular diseases using data from about 60,000 individuals (24). siRNA therapeutics, including lepodisiran, olpasiran, and zerlasiran (SLN360), lowered Lp(a) by >95% at higher doses (16, 18, 26). Pelacarsen, an anti-sense oligonucleotide, reduced Lp(a) by 70%–80% at 60 mg every 4 weeks and 20 mg once weekly (17) and had a reported 92% reduction in the 40 mg multiple ascending dose cohort in an earlier phase I/IIa trial (27). Our current study demonstrated that muvalaplin achieved 70%–80% lowering at a once-daily dose of 300 mg, 500 mg, and 800 mg, when measured using the intact Lp(a) assay. Although these molecules in development can reduce Lp(a) levels significantly, their therapeutic benefits in reducing cardiovascular morbidity and mortality events remain to be demonstrated by outcome trials.

Future efforts will include studying the kinetics and the effects of unbound apo(a) that could be present as apo(a) or apo(a)-muvalaplin complexes. Previous studies examining the existence of free apo(a) in human plasma have shown that free apo(a) constitutes only 3%–5% of the total Lp(a), with the vast majority of the free apo(a) present as degradation fragments (28, 29). The pathogenicity of free apo(a) versus Lp(a) is not clear, although many studies have shown that pathological effects of Lp(a) in in vitro model systems can be recapitulated by apo(a) (30). Nonetheless, mixed results have been observed in experimental apo(a) and Lp(a) animal models of atherosclerosis (31). Therefore, whether Lp(a) or free apo(a) have pathological functions that are unique to them is not known.

Our study has limitations. Clinical data linking Lp(a) to clinical outcomes is largely based on measuring total apo(a). Additional studies are needed to compare the ability of an intact Lp(a) assay to predict these clinical outcomes. The muvalaplin phase I study was small, had a relatively low inclusion criterion for Lp(a) (≥30 mg/dl), and had a treatment period of only 14 days. The study population was generally healthy. Accordingly, this study did not include individuals with cardiovascular disease, renal impairment, hepatic dysfunction, or identified significant mutations of the *LPA* gene. Finally, whether Lp(a) lowering with muvalaplin, or any Lp(a) targeted therapy, will reduce cardiovascular risk remains to be studied by cardiovascular outcome trials.

## Conclusion

Lp(a) has been recognized and studied as an independent cardiovascular risk factor and studied as a therapeutic target to reduce the incidence of major adverse cardiovascular events. Accurate measurement of the Lp(a) concentration is challenging because of the heterogenicity of Lp(a) particles and potential interference from unbound apo(a). Since commercially available assays measure total apo(a), a novel isoform-independent intact Lp(a) particle-specific immunoassay was developed to measure intact Lp(a). Results with this intact Lp(a) assay revealed the potent Lp(a)-lowering effect of muvalaplin, a novel small molecule that disrupts the binding of apo(a) to apoB. This should aid in the more accurate development of novel Lp(a) targeted therapeutics that aim to reduce the burden of cardiovascular diseases.

## Data availability

All data are contained within the manuscript.

## Supplemental data

This article contains supplemental data.

## Conflict of interest

The authors declare the following financial interests/personal relationships which may be considered as potential competing interests: Mr. Swearingen and Drs Sloan, Rhodes, Siegel, Bivi, Qian, Konrad, Krege, Ruotolo, Michael, and Wen are employees of and stockholders in Eli Lilly and Company. Dr Boffa is an employee at The University of Western Ontario, has received funding from the 10.13039/501100000024Canadian Institutes of Health Research and the Heart and Stroke Foundation of Ontario, and is a consultant for Eli Lilly and Company. Dr Koschinsky is an employee at the University of Western Ontario, has received funding from the 10.13039/501100000024Canadian Institutes of Health Research and the Heart and Stroke Foundation of Ontario, is a consultant or on the advisory board for Novartis Canada and Eli Lilly and Company, and has research contracts with Abcentra and Eli Lilly and Company. Dr Nicholls has received research support from 10.13039/100004325AstraZeneca, 10.13039/100002429Amgen, 10.13039/100014929Anthera, 10.13039/100008322CSL Behring, Cerenis, Eli Lilly, 10.13039/501100022336Esperion, Resverlogix, 10.13039/100004336Novartis, 10.13039/100018503InfraReDx, and Sanofi-Regeneron and is a consultant for Amgen, Akcea, AstraZeneca, Boehringer Ingelheim, CSL Behring, Daiichi Sankyo, Eli Lilly, Esperion, Kowa, Merck, Takeda, Pfizer, Sanofi-Regeneron, Novo Nordisk, CSL Sequiris, and Vaxxinity.

## References

[1] Wulff A.B., Nordewstgaard B.G., Langsted A. (2024). Novel therapies for lipoprotein(a): update in cardiovascular risk estimation and treatment. Curr. Atheroscler. Rep..

[2] Boffa M.B., Koschinsky M.L. (2022). Understanding the ins and outs of lipoprotein(a) metabolism. Curr. Opin. Lipidol..

[3] Reyes-Soffer G., Ginsberg H.N., Berglund L., Barton Duell P., Heffron S.P., Kamstrup P.R. (2022). Lipoprotein(a): a genetically determined, causal, and prevalent risk factor for atherosclerotic cardiovascular disease: a scientific statement from the American Heart Association. Arterioscler. Thromb. Vasc. Biol..

[4] Kronenberg F., Mora S., Stroes E.S.G., Ference B.A., Arsenault B.J., Berglund L. (2022). Lipoprotein(a) in atherosclerotic cardiovascular disease and aortic stenosis: a European Atherosclerosis Society consensus statement. Eur. Heart J..

[5] Schmidt K., Noureen A., Kronenberg F., Utermann G. (2016). Structure, function, and genetics of lipoprotein (a). J. Lipid Res..

[6] Coassin S., Kronenberg F. (2022). Lipoprotein(a) beyond the kringle IV repeat polymorphism: the complexity of genetic variation in the LPA gene. Atherosclerosis.

[7] Boerwinkle E., Leffert C.C., Lin J., Lackner C., Chiesa G., Hobbs H.H. (1992). Apolipoprotein(a) gene accounts for greater than 90% of the variation in plasma lipoprotein(a) concentrations. J. Clin. Invest..

[8] Marcovina S.M., Albers J.J., Gabel B., Koschinsky M.L., Gaur V.P. (1995). Effect of the number of apolipoprotein(a) kringle 4 domains on immunochemical measurements of lipoprotein(a). Clin. Chem..

[9] Marcovina S.M., Albers J.J. (2016). Lipoprotein (a) measurements for clinical application. J. Lipid Res..

[10] Wyness S.P., Genzen J.R. (2021). Performance evaluation of five lipoprotein(a) immunoassays on the Roche cobas c501 chemistry analyzer. Pract. Lab. Med..

[11] Scharnagl H., Stojakovic T., Dieplinger B., Dieplinger H., Erhart G., Kostner G.M. (2019). Comparison of lipoprotein (a) serum concentrations measured by six commercially available immunoassays. Atherosclerosis.

[12] Marcovina S.M., Navabi N., Allen S., Gonen A., Witztum J.L., Tsimikas S. (2022). Development and validation of an isoform-independent monoclonal antibody-based ELISA for measurement of lipoprotein(a). J. Lipid Res..

[13] Tsimikas S., Lau H.K., Han K.-R., Shortal B., Miller E.R., Segev A. (2004). Percutaneous coronary intervention results in acute increases in oxidized phospholipids and lipoprotein(a): short-term and long-term immunologic responses to oxidized low-density lipoprotein. Circulation.

[14] Verbeek R., Boekholdt S.M., Stoekenbroek R.M., Kees Hovingh G., Witztum J.L., Wareham N.J. (2016). Population and assay thresholds for the predictive value of lipoprotein (a) for coronary artery disease: the EPIC-Norfolk Prospective Population Study. J. Lipid Res..

[15] Trenkwalder E., Gruber A., König P., Dieplinger H., Kronenberg F. (1997). Increased plasma concentrations of LDL-unbound apo(a) in patients with end-stage renal disease. Kidney Int..

[16] O'Donoghue M.L., Rosenson R.S., Gencer B., López J.A.G., Lepor N.E., Baum S.J. (2022). Small interfering RNA to reduce lipoprotein(a) in cardiovascular disease. N. Engl. J. Med..

[17] Tsimikas S., Karwatowska-Prokopczuk E., Gouni-Berthold I., Tardif J.-C., Baum S.J., Steinhagen-Thiessen E. (2020). Lipoprotein(a) reduction in persons with cardiovascular disease. N. Engl. J. Med..

[18] Nissen S.E., Linnebjerg H., Shen X., Wolski K., Ma X., Lim S. (2023). Lepodisiran, an extended-duration short interfering RNA targeting lipoprotein(a): a randomized dose-ascending clinical trial. JAMA.

[19] Nicholls S.J., Nissen S.E., Gleming C., Urva S., Suico J., Berg P.H. (2023). Muvalaplin, an oral small molecule inhibitor of lipoprotein(a) formation: a randomized clinical trial. JAMA.

[20] Diaz N., Perez C., Escribano A.M., Sanz G., Priego J., Lafuente C. (2024). Discovery of potent small-molecule inhibitors of lipoprotein(a) formation. Nature.

[21] Marcovina S.M., Clouet-Foraison N., Koschinsky M.L., Lowenthal M.S., Orquillas A., Boffa M.B. (2021). Development of an LC-MS/MS proposed candidate reference method for the standardization of analytical methods to measure lipoprotein(a). Clin. Chem..

[22] Kronenberg F. (2019). Therapeutic lowering of lipoprotein(a): how much is enough?. Atherosclerosis.

[23] Lamina C., Kronenberg F., Lp(a)-GWAS-Consortium (2019). Estimation of the required lipoprotein(a)-lowering therapeutic effect size for reduction in coronary heart disease outcomes: a Mendelian randomization analysis. JAMA Cardiol..

[24] Madsen C.M., Kamstrup P.R., Langsted A., Varbo A., Nordestgaard B.G. (2020). Lipoprotein(a)-lowering by 50 mg/dL (105 nmol/L) may be needed to reduce cardiovascular disease 20% in secondary prevention: a population-based study. Arterioscler. Thromb. Vasc. Biol..

[25] Burgess S., Ference B.A., Staley J.R., Freitag D.F., Mason A.M., Nielsen S.F. (2018). Association of LPA variants with risk of coronary disease and the implications for lipoprotein(a)-lowering therapies: a Mendelian randomization analysis. JAMA Cardiol..

[26] Nissen S.E., Wolski K., Balog C., Swerdlow D.I., Scrimgeour A.C., Rambaran C. (2022). Single ascending dose study of a short interfering RNA targeting lipoprotein(a) production in individuals with elevated plasma lipoprotein(a) levels. JAMA.

[27] Viney N.J., van Capelleveen J.C., Geary R.S., Xia S., Tami J.A., Yu R.Z. (2016). Antisense oligonucleotides targeting apolipoprotein(a) in people with raised lipoprotein(a): two randomised, double-blind, placebo-controlled, dose-ranging trials. Lancet.

[28] Gries A., Nimpf J., Nimpf M., Wurm H., Kostner G.M. (1987). Free and Apo B-associated Lpa-specific protein in human serum. Clin. Chim. Acta.

[29] Mooser V., Marcovina S.M., White A.L., Hobbs H.H. (1996). Kringle-containing fragments of apolipoprotein(a) circulate in human plasma and are excreted into the urine. J. Clin. Invest..

[30] Boffa M.B., Koschinsky M.L. (2024). Lipoprotein(a) and cardiovascular disease. Biochem. J..

[31] Yeang C., Cotter B., Tsimikas S. (2016). Experimental animal models evaluating the causal role of lipoprotein(a) in atherosclerosis and aortic stenosis. Cardiovasc. Drugs Ther..

