# Supplementary material for: Measuring Lp(a) particles with a novel isoform-insensitive immunoassay illustrates efficacy of muvalaplin
Source: J Lipid Res. 2024 Dec 6;66(1):100723. doi: 10.1016/j.jlr.2024.100723 (PMC11761857; doi:10.1016/j.jlr.2024.100723)
Supplement: Supplemental information [file mmc1.docx]

**Measuring Lp(a) particles with a novel isoform-insensitive immunoassay illustrates efficacy of muvalaplin**

Craig A. Swearingen^1^, John H. Sloan^1^, Grace M. Rhodes^1^, Robert W. Siegel^1^, Nico Bivi^1^, Yuewei Qian^1^, Robert J. Konrad^1^, Michael Boffa^2^, Marlys Koschinsky^2^, John Krege^1^, Giacomo Ruotolo^1^, Stephen J. Nicholls^3^, Laura F. Michael^1^*, and Yi Wen^1^*

^1^Lilly Research Laboratories, Eli Lilly and Company, Indianapolis, IN, USA 46285

^2^Robarts Research Institute, University of Western Ontario, London, Ontario, Canada N6A 5B7

^3^Victorian Heart Institute, Monash University, Clayton, VIC, Australia

*Corresponding authors: Laura Michael (Michael_laura@lilly.com) and Yi Wen (wen_yi1@lilly.com)

**Table S1: Standard curve of the intact lipoprotein(a) [Lp(a)] assay**

| Theoretical | | ECLU | | | Back-calculated concentration | | | |
| --- | --- | --- | --- | --- | --- | --- | --- | --- |
| mg/dL | **nM** | **Mean** | **SD** | **%CV** | **Mean, nM** | **%Change from theoretical** | **SD** | **%CV** |
| 0.5 | 0.925 | 288,791 | 8,370.1 | 2.9 | 0.957665 | 3.5 | 0.01229 | 1.3 |
| 0.166667 | 0.3083333 | 155,098 | 5,452 | 3.5 | 0.300833 | −2.4 | 0.00346 | 1.1 |
| 0.055556 | 0.1027778 | 67,137 | 2,832.3 | 4.2 | 0.100143 | −2.6 | 0.00082 | 0.8 |
| 0.018519 | 0.0342593 | 26,161 | 1,175.4 | 4.5 | 0.034517 | 0.8 | 0.00023 | 0.7 |
| 0.006173 | 0.0114198 | 9,398 | 455.5 | 4.8 | 0.011568 | 1.3 | 0.00012 | 1.1 |
| 0.002058 | 0.0038066 | 3,336 | 176.9 | 5.3 | 0.003891 | 2.2 | 0.00003 | 0.7 |
| 0.000686 | 0.0012689 | 1,173 | 61 | 5.2 | 0.001278 | 0.7 | 0.00002 | 1.3 |
| 0.000229 | 0.000423 | 439 | 23.3 | 5.3 | 0.000423 | 0.0 | 0.00001 | 2.4 |
| 0.000076 | 0.000141 | 185 | 8.1 | 4.4 | 0.000137 | −2.8 | 0.00000 | 2.1 |
| 0.000025 | 0.000047 | 102 | 3.9 | 3.8 | 0.000046 | −1.6 | 0.00000 | 3.8 |
| 0.000008 | 0.000016 | 74 | 2.5 | 3.4 | 0.000016 | −0.7 | 0.00000 | 11.6 |
| 0 | 0 | 59 | 3.5 | 5.9 | NA | NA | NA | NA |

In total, 64 sets of standard curves were collected over 30 days. The mean, standard deviation (SD), and % coefficient of variation (CV) of the electrochemiluminescent unit (ECLU) signals and back-calculated values at each standard level, and percent change from theoretical value were calculated. NA, not applicable.

**Table S2: Lipoprotein(a) levels in 60 healthy human samples**

| Sample | Run 1 | Run 2 | Mean | SD | %CV | Sample | Run 1 | Run 2 | Mean | SD | %CV |
| --- | --- | --- | --- | --- | --- | --- | --- | --- | --- | --- | --- |
| 1 | 99.2 | 102.5 | 100.9 | 2.4 | 2.3 | 31 | 57.8 | 60.1 | 58.9 | 1.7 | 2.8 |
| 2 | 24.7 | 26.6 | 25.7 | 1.3 | 5.2 | 32 | 43.2 | 43.2 | 43.2 | 0.0 | 0.0 |
| 3 | 28.3 | 28.0 | 28.2 | 0.2 | 0.8 | 33 | 34.9 | 34.6 | 34.7 | 0.2 | 0.6 |
| 4 | 15.4 | 14.7 | 15.0 | 0.5 | 3.5 | 34 | 9.1 | 9.7 | 9.4 | 0.4 | 4.7 |
| 5 | 61.1 | 62.1 | 61.6 | 0.7 | 1.1 | 35 | 232.8 | 239.6 | 236.2 | 4.8 | 2.0 |
| 6 | 9.3 | 10.5 | 9.9 | 0.9 | 8.7 | 36 | 15.9 | 17.6 | 16.7 | 1.2 | 7.4 |
| 7 | 10.6 | 12.2 | 11.4 | 1.2 | 10.2 | 37 | 38.6 | 39.2 | 38.9 | 0.4 | 1.1 |
| 8 | 50.8 | 56.5 | 53.6 | 4.0 | 7.5 | 38 | 93.6 | 93.3 | 93.5 | 0.2 | 0.2 |
| 9 | 14.2 | 15.1 | 14.6 | 0.7 | 4.6 | 39 | 26.4 | 25.5 | 26.0 | 0.6 | 2.3 |
| 10 | 113.0 | 120.9 | 116.9 | 5.6 | 4.8 | 40 | 13.1 | 12.3 | 12.7 | 0.6 | 4.7 |
| 11 | 122.8 | 141.7 | 132.3 | 13.4 | 10.1 | 41 | 99.9 | 104.6 | 102.3 | 3.3 | 3.2 |
| 12 | 41.4 | 43.9 | 42.6 | 1.8 | 4.2 | 42 | 232.1 | 238.6 | 235.4 | 4.6 | 1.9 |
| 13 | 156.5 | 159.2 | 157.8 | 1.9 | 1.2 | 43 | 47.9 | 52.4 | 50.2 | 3.2 | 6.4 |
| 14 | 11.2 | 11.5 | 11.4 | 0.2 | 1.8 | 44 | 16.9 | 18.6 | 17.7 | 1.2 | 6.8 |
| 15 | 59.5 | 61.4 | 60.5 | 1.3 | 2.2 | 45 | 56.1 | 58.1 | 57.1 | 1.4 | 2.5 |
| 16 | 25.4 | 26.3 | 25.8 | 0.6 | 2.5 | 46 | 91.8 | 98.0 | 94.9 | 4.4 | 4.6 |
| 17 | 9.9 | 9.3 | 9.6 | 0.4 | 4.5 | 47 | 20.1 | 20.9 | 20.5 | 0.5 | 2.6 |
| 18 | 26.2 | 26.2 | 26.2 | 0.0 | 0.1 | 48 | 66.0 | 71.4 | 68.7 | 3.8 | 5.5 |
| 19 | 87.5 | 88.2 | 87.9 | 0.5 | 0.6 | 49 | 33.3 | 35.5 | 34.4 | 1.6 | 4.5 |
| 20 | 41.4 | 44.6 | 43.0 | 2.3 | 5.3 | 50 | 17.0 | 17.8 | 17.4 | 0.6 | 3.2 |
| 21 | 47.9 | 61.8 | 54.8 | 9.8 | 17.8 | 51 | 226.2 | 239.2 | 232.7 | 9.2 | 4.0 |
| 22 | 6.4 | 7.4 | 6.9 | 0.8 | 10.9 | 52 | 6.5 | 7.2 | 6.8 | 0.5 | 7.5 |
| 23 | 53.4 | 58.2 | 55.8 | 3.4 | 6.2 | 53 | 101.8 | 110.6 | 106.2 | 6.2 | 5.9 |
| 24 | 27.3 | 29.4 | 28.4 | 1.5 | 5.3 | 54 | 9.6 | 9.6 | 9.6 | 0.0 | 0.3 |
| 25 | 61.1 | 65.2 | 63.2 | 2.9 | 4.6 | 55 | 20.1 | 21.0 | 20.5 | 0.6 | 3.1 |
| 26 | 200.8 | 193.9 | 197.4 | 4.9 | 2.5 | 56 | 89.9 | 92.6 | 91.3 | 1.9 | 2.1 |
| 27 | 99.1 | 103.9 | 101.5 | 3.4 | 3.4 | 57 | 189.9 | 203.0 | 196.5 | 9.3 | 4.7 |
| 28 | 46.3 | 49.5 | 47.9 | 2.3 | 4.7 | 58 | 45.5 | 47.6 | 46.5 | 1.5 | 3.2 |
| 29 | 138.6 | 146.4 | 142.5 | 5.5 | 3.9 | 59 | 107.5 | 108.6 | 108.0 | 0.8 | 0.7 |
| 30 | 33.5 | 35.4 | 34.5 | 1.4 | 4.0 | 60 | 89.4 | 95.8 | 92.6 | 4.5 | 4.9 |

Lipoprotein(a) in 60 healthy human samples were measured in two independent runs on 2 separate days. CV: coefficient of variation; SD: standard deviation.

**Table S3: Lp(a) measurements in the presence of muvalaplin**

|  | % Recovery compared to 0 ng/mL muvalaplin | | | |
| --- | --- | --- | --- | --- |
|  | 250 ng/mL | 125 ng/mL | 62.5 ng/mL | 31.25 ng/mL |
| Serum 1, 180.8 nM | 97.1% | 98.8% | 107.3% | 101.5% |
| Serum 2, 43.0 nM | 95.3% | 109.4% | 107.4% | 103.0% |
| Serum 3, 12.1 nM | 89.6% | 100.9% | 105.4% | 102.3% |
| Average | 94.0% | 103.1% | 106.7% | 102.3% |

Three human serum samples of different Lp(a) levels were spiked with up to 250 ng/mL muvalaplin. The measured Lp(a) levels in the spiked samples were compared to corresponding serum samples without muvalaplin spike (0 ng/mL muvalaplin).


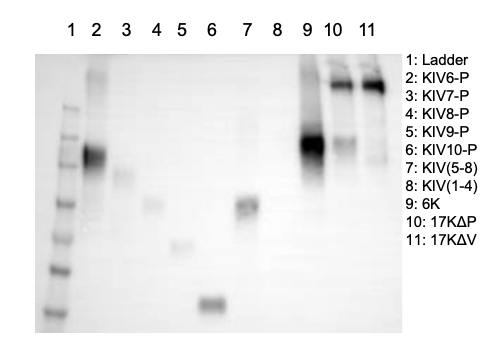


**Figure S1: Binding specificity of the anti-apolipoprotein(a) [apo(a)] capture antibody by Western blotting**

Each recombinant apo(a) protein variant (0.25 µg) was subjected to SDS-PAGE on a 4–15% polyacrylamide gradient gel, followed by immunoblotting using the capture antibody (0.5 µg/mL) and a horseradish peroxidase-linked sheep anti-mouse immunoglobulin secondary antibody. Different recombinant apo(a) protein variants were produced and named based on 17K, the parental, full-length recombinant apo(a) protein. The “P” denotes the protease domain. KIV6-P, KIV7-P, KIV8-P, KIV9-P, KIV10-P, KIV(5-8), and KIV(1-4), were named with starting domain and end domain. The 6K protein contains a KIV1/KIV5 hybrid kringle and KIV6-P. 17KΔP is the 17K protein without protease domain. 17KΔV is the 17K protein without KV domain.


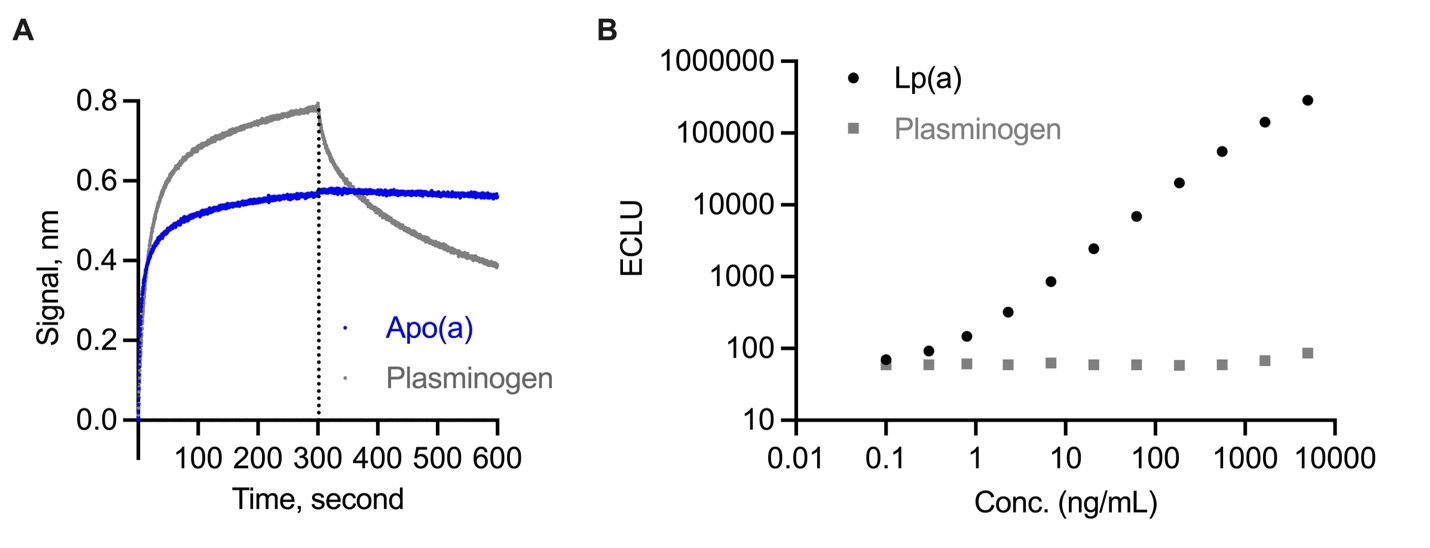


**Figure S2: Specificity of the anti-apo(a) antibody and intact Lp(a) assay to plasminogen**

A, binding of the anti-apo(a) capture antibody to apo(a) and plasminogen was studied by bio-layer interferometry. B, standard curve of the intact Lp(a) assay using Lp(a) or plasminogen as the standard.


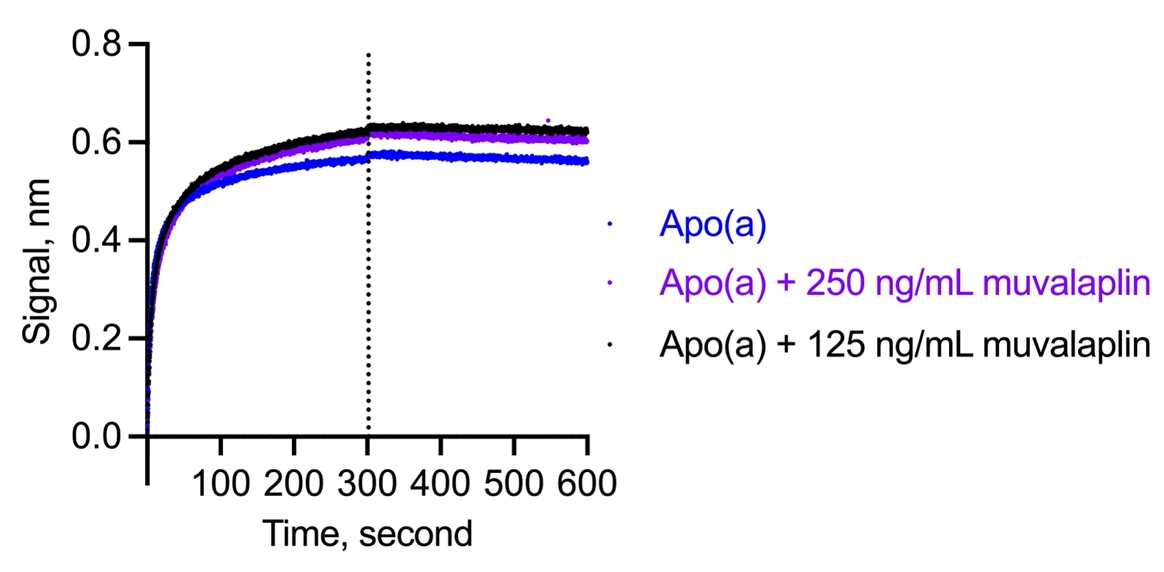


**Figure S3: Binding of anti-apo(a) antibody to apo(a) in the presence of muvalaplin**

Binding of the anti-apo(a) capture antibody to apo(a) in the presence of 250 ng/mL or 125 ng/mL of muvalaplin was studied by bio-layer interferometry.
